# Development of Guidelines to Inform the Introduction of Exercise in Young People With Eating Disorders

**DOI:** 10.1192/bjo.2026.11492

**Published:** 2026-06-30

**Authors:** Jonathan Stott, Gareth Howel, Rebecca Ferris, Hannah Townsend, Susan Calvert

**Affiliations:** 1 Tees, Esk and Wear Valleys NHS Foundation Trust, Northallerton, United Kingdom; 2 Tees, Esk and Wear Valleys NHS Foundation Trust, York, United Kingdom; 3 Tees, Esk and Wear Valleys NHS Foundation Trust, Harrogate, United Kingdom; 4 Hull University Teaching Hospital NHS, Hull, United Kingdom

## Abstract

**Aims:**

Tees, Esk and Wear Valley’s Eating Disorder teams recognised a lack of guidance to inform the introduction of exercise in young people with eating disorders. An MDT group reviewed evidence and international guidelines to inform the drafting of a decision-making tool in line with national risk assessment frameworks.

**Methods:**

1) A multidisciplinary team assembled monthly, including physical and mental health clinicians and dieticians, to develop and refine guidelines.

2) A literature search was conducted to seek up to date best practice globally.

3) The impact and utility of the framework was assessed through pre-and post-guideline questionnaires distributed to the Eating Disorder Team.

4) Artificial Intelligence assisted Thematic Analysis explored pre-and post-guideline responses.

**Results:**

Thematic Analysis highlighted:

1. Guidance and decision tree were seen as helpful and providing more structure. There were still some requests for improved communication and shared application.

2. Some variation in decision making exists post guidelines, but several team members noted improved consistency with guidance/decision tools. Some pockets of inconsistency remain.

3. Greater reflection on balance of risk. Some felt the team were still overly cautious in the reintroduction of exercise, but others noted guidance enabled positive risk-taking for young people.

4. Confidence generally higher in this area within the team. Respondents highlighted MDT strength and guidance as supportive tools. Some newer staff still unsure around advice in this area.

**Conclusion:**

Themes emergent from pre-and post-draft guideline questionnaires reflected those identified in wider clinical studies around the management of exercise in Eating Disorder interventions. Teams believed exercise was crucial to recovery, bringing benefits to mental and physical health, providing connection with past activities that previously brought enjoyment. The introduction of new clinical guidelines were felt to enable positive risk taking whilst improving consistency and confidence within the MDT.

